# Structural insights into the regulation of SigB activity by RsbV and RsbW

**DOI:** 10.1107/S2052252520007617

**Published:** 2020-06-24

**Authors:** Deepak Pathak, Kyeong Sik Jin, Sudarshan Tandukar, Jun Ha Kim, Eunju Kwon, Dong Young Kim

**Affiliations:** a College of Pharmacy, Yeungnam University, Gyeongsan, Gyeongbuk 38541, Republic of Korea; b Pohang Accelerator Laboratory (PAL), Pohang University of Science and Technology, Pohang, Gyeongbuk 37673, Republic of Korea

**Keywords:** sigma factor, anti-sigma factor, anti-anti-sigma factor, crystal structure, small-angle X-ray scattering

## Abstract

*Bacillus subtilis* SigB is an alternative sigma factor that initiates the transcription of stress-responsive genes. The anti-sigma factor RsbW tightly binds SigB to suppress its activity under normal growth conditions and releases it when nonphosphorylated RsbV binds to RsbW in response to stress signals. To understand the regulation of SigB activity by RsbV and RsbW based on structural features, crystal structures and a small-angle X-ray scattering (SAXS) envelope structure of the RsbV–RsbW complex were determined. The crystal structures showed that RsbV and RsbW form a heterotetramer in a similar manner to a SpoIIAA–SpoIIAB tetramer. Multi-angle light scattering and SAXS revealed that the RsbV–RsbW complex is an octamer in solution. Superimposition of the crystal structure on the SAXS envelope structure showed that the unique dimeric interface of RsbW mediates the formation of an RsbV–RsbW octamer and does not prevent RsbV and SigB from binding to RsbW. These results provide structural insights into the molecular assembly of the RsbV–RsbW complex and the regulation of SigB activity.

## Abstract 2

Crystal structures and a SAXS envelope model of the RsbV–RsbW complex reveal that the RsbW tetramer forms a core structure to regulate SigB activity.

## 1. Introduction   

Bacterial sigma factors recognize promoter elements and initiate transcription as key components of the bacterial RNA polymerase holoenzyme (Burgess *et al.*, 1969 ▸; Saecker *et al.*, 2011 ▸). Almost all bacteria harbor multiple sigma factors, each of which regulates the transcription of a group of genes known as a regulon, depending on its promoter-sequence preferences (Gross *et al.*, 1998 ▸; Wösten, 1998 ▸). To regulate the activity of sigma factors, anti-sigma factors suppress their partner sigma factors under normal growth conditions and release the sigma factors in response to specific signals; this differs for primary sigma factors, which initiate the transcription of housekeeping genes required for cellular maintenance under normal growth conditions (Paget, 2015 ▸).

Various mechanisms have been described in which anti-sigma factors suppress sigma factors and release them in response to stimuli (Paget, 2015 ▸). Many anti-sigma factors, including those for *Escherichia coli* SigE and *Bacillus subtilis* SigW, are degraded by regulated intramembrane proteolysis in response to extracellular signals, releasing their associated sigma factors (Barchinger & Ades, 2013 ▸; Heinrich *et al.*, 2009 ▸). Some anti-sigma factors, such as those for *E. coli* Sig32 and *Streptomyces coelicolor* SigR, unleash sigma factors by changing their own conformation directly in response to stress signals (Chakraborty *et al.*, 2014 ▸; Rajasekar *et al.*, 2016 ▸; Chattopadhyay & Roy, 2002 ▸). Some anti-sigma factors, such as those for *B. subtilis* SigF and SigB, release their sigma factors by exchanging binding partners with anti-anti-sigma factors (Duncan *et al.*, 1996 ▸; Magnin *et al.*, 1997 ▸).

**Figure 1:**
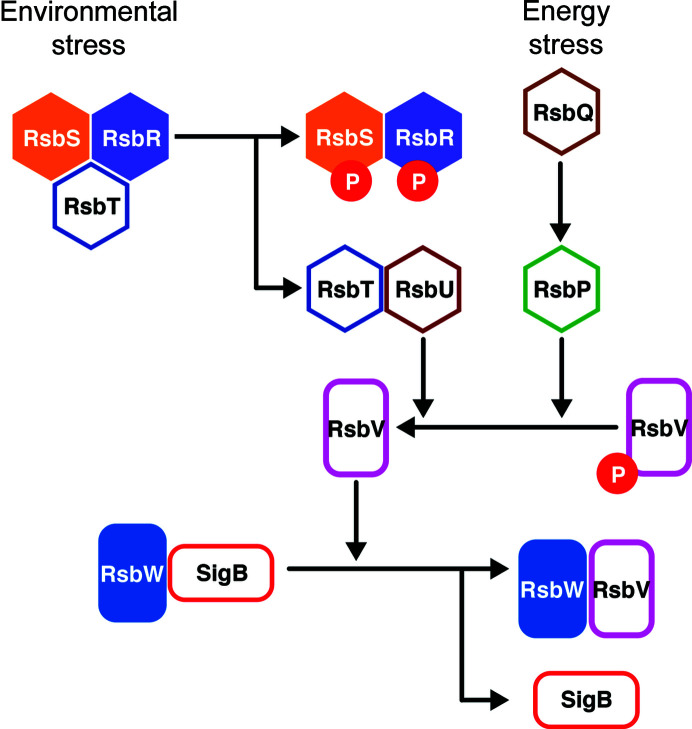
Schematic of the SigB activation signaling pathway. SigB is released from RsbW in response to environmental and energy stresses, initiating signal transduction.

The anti-sigma factor RsbW releases SigB to induce SigB-dependent transcription under specific stress conditions. More than 150 genes are transcribed by the SigB-associated RNA polymerase under diverse signals such as energy starvation, heat, salt and ethanol (Hecker & Völker, 2001 ▸; Hecker *et al.*, 2007 ▸). Although energy and environmental stress signals activate different signaling pathways, both of the pathways commonly regulate the binding of the anti-anti-sigma factor RsbV to RsbW through the dephosphorylation of RsbV (Voelker *et al.*, 1995 ▸, 1996 ▸; Fig. 1 ▸). In these signaling pathways, energy stress activates the phosphatase RsbP via RsbQ (Vijay *et al.*, 2000 ▸; Brody *et al.*, 2001 ▸), while environmental stressors activate the phosphatase RsbU via a stressosome, a large protein complex composed of RsbS, RsbT and RsbR (Chen *et al.*, 2003 ▸; Kuo *et al.*, 2004 ▸). RsbP and RsbU dephos­phorylate RsbV under stress conditions; RsbV then tightly binds to RsbW and triggers the release of SigB (Dufour & Haldenwang, 1994 ▸; Voelker *et al.*, 1996 ▸). Conversely, in the absence of stress signals, RsbW phosphorylates RsbV and binds to SigB (Dufour & Haldenwang, 1994 ▸). That is, SigB activity is determined by the presence of the dominant kinase (RsbW) or phosphatase (RsbU and RsbP), which is determined by the environmental conditions (Voelker *et al.*, 1995 ▸; Dufour & Haldenwang, 1994 ▸; Voelker *et al.*, 1996 ▸). To understand the structural basis of how SigB activity is regulated by the anti-sigma factor RsbW and the anti-anti-sigma factor RsbV, we analyzed the molecular assembly of the RsbV–RsbW complex using multi-angle light scattering (MALS) and small-angle X-ray scattering (SAXS) envelope structures as well as the crystal structures.

## 2. Experimental procedures   

### 2.1. Plasmid preparation, protein expression and purification   

Genes encoding RsbV (residues 1–109), RsbW (residues 1–160) and SigB (residues 1–262) were amplified by polymerase chain reaction from the genomic DNA of *B. subtilis* strain 168. DNA encoding a 6×His-thioredoxin-TEV protease cleavage site was pre-inserted upstream of the multi-cloning site (MCS) in the pETDuet-1 and pET-28b vectors (Merck Millipore, Billerica, Massachusetts, USA). A plasmid encoding both RsbV and RsbW was prepared by inserting *rsbV* and *rsbW* into MCS-1 and MCS-2, respectively, of the modified pETDuet-1 vector. The plasmid encoding SigB and RsbW was prepared by inserting *rsbW* and *sigB* into the modified pETDuet-1. Plasmids encoding RsbV or RsbW alone were prepared by inserting *rsbV* or *rsbW* into the modified pET-28b vector.

RsbW and 6×His-thioredoxin-RsbV were expressed in *E. coli* strain BL21 Star (DE3) cells (Thermo Fisher Scientific, Waltham, Massachusetts, USA). The *E. coli* cells were transformed with the plasmid encoding both RsbV and RsbW and were cultured in Luria–Bertani medium at 37°C. When the optical density at 600 nm reached 0.6–0.7, protein expression was induced by adding 0.4 m*M* isopropyl β-d-1-thio­galacto­pyran­oside. After overnight culture at 20°C, the cells were harvested by centrifugation at 3000*g* for 10 min, resuspended in buffer *A* (20 m*M* HEPES pH 7.5, 0.2 *M* NaCl, 0.2 m*M* TCEP, 5% glycerol) and disrupted by sonication. Cell lysates were incubated with DNase I (Roche, Mannheim, Germany) and RNase A (Roche) at a concentration of 10 µg ml^−1^ for 30 min and then clarified by centrifugation at 20 000*g*. The RsbV–RsbW complex was purified by immobilized metal-affinity chromatography and size-exclusion chromatography (SEC). The clarified cell lysates were loaded onto a 5 ml HisTrap chelating column charged with nickel ion (GE Healthcare Life Sciences, Uppsala, Sweden). Proteins bound to the column were eluted with a concentration gradient of 0.05–0.5 *M* imidazole. Fractions containing 6×His-thioredoxin-RsbV and RsbW were pooled and treated with TEV protease to isolate 6×His-thioredoxin and RsbV. After complete cleavage, the protein solution was dialyzed in buffer *A* and was subjected to a nickel-charged resin to remove 6×His-thio­redoxin. Next, the RsbV–RsbW complex was purified by two rounds of SEC using a Superdex 200 preparatory-grade column (GE Healthcare Life Sciences) pre-equilibrated with buffer *A*. Truncated RsbV–RsbW complexes and RsbW–SigB were expressed and purified as described above for full-length RsbV and RsbW. RsbV and RsbW alone were similarly expressed and purified following the same procedures, except that Superdex 75 preparatory-grade columns were used for SEC (GE Healthcare Life Sciences). For MALS and SAXS experiments, protein buffers were exchanged by SEC using a Superdex 200 analytical column (GE Healthcare Life Sciences).

### 2.2. Crystallization, data collection and structure determination   

Crystals of a truncated RsbV–RsbW complex (RsbV_1–104_–RsbW_5–145_) that were suitable for X-ray data collection were grown in micro-batch plates at 20°C. The crystallization drop was prepared by mixing 1 µl protein solution (10 mg ml^−1^) with 1 µl crystallization solution under a layer of Al’s oil (Hampton Research, Aliso Viejo, California, USA). Plate-shaped monoclinic crystals of RsbV_1–104_–RsbW_5–145_ grew completely within three weeks in a crystallization solution consisting of 25%(*w*/*v*) PEG 1500, 100 m*M* SPG buffer pH 8.5 [Supplementary Fig. S1 ▸(*b*)]. Rod-shaped hexagonal crystals of RsbV_1–104_–RsbW_5–145_ grew in a crystallization solution consisting of 20%(*w*/*v*) PEG 3350, 200 m*M* potassium formate in the presence of excess ADP [Supplementary Fig. S1 ▸(*c*)]. Single crystals were picked up with a CryoLoop (Hampton Research) without the addition of cryoprotectant and rapidly cooled in a cold nitrogen stream. Diffraction data were collected on beamline 11C at Pohang Light Source II, Republic of Korea (PLS II-BL11C; Park *et al.*, 2017 ▸) with a PILATUS3 6M detector (Dectris, Baden-Daettwil, Switzerland) and were indexed, integrated and scaled using *HKL*-2000 (Otwinowski & Minor, 1997 ▸).

The monoclinic crystal structure of RsbV_1–104_–RsbW_5–145_ was determined by the molecular-replacement (MR) method. Homology models of RsbV and RsbW were created from the structures of SpoIIAA (PDB entry 1thn; Masuda *et al.*, 2004 ▸) and SpoIIAB (PDB entry 1til; Masuda *et al.*, 2004 ▸) respectively, using the *SWISS-MODEL* server (Guex *et al.*, 2009 ▸). Next, the homology models were superimposed on the structure of the SpoIIAA–SpoIIAB complex (PDB entry 1til) to generate a model structure of the RsbV–RsbW complex. Initially, four RsbV–RsbW dimers were found by *Phaser* (McCoy *et al.*, 2007 ▸) using a truncated model of the RsbV–RsbW dimer. A total of eight RsbV–RsbW dimers were built in the asymmetric unit. Model building and structure refinement were performed using *phenix.refine* (Afonine *et al.*, 2012 ▸) and *Coot* (Emsley *et al.*, 2010 ▸). The final model of the monoclinic crystal structure was refined to *R* and *R*_free_ values of 20.8% and 29.0%, respectively, at 3.4 Å resolution. The hexagonal crystal structure of RsbV_1–104_–RsbW_5–145_ was determined by MR using an RsbV–RsbW tetramer from the monoclinic crystal structure as a template. This final model was refined to *R* and *R*_free_ values of 22.1% and 24.9%, respectively, at 3.1 Å resolution. The data-collection and refinement statistics are summarized in Table 1 ▸. Structural alignment, protein–protein interactions and surface area were analyzed using the *DALI* server (Holm & Laakso, 2016 ▸), *LIGPLOT* (Laskowski & Swindells, 2011 ▸) and *PDBePISA* (Krissinel & Henrick, 2007 ▸), respectively. Figures were drawn using *PyMOL* (version 1.8; Schrödinger) and *ALSCRIPT* (Barton, 1993 ▸). The final coordinates and structure factors were deposited in the Protein Data Bank with PDB codes 6m36 for the monoclinic crystal structure and 6m37 for the hexagonal crystal structure.

### 2.3. Asymmetrical flow-field flow fractionation with MALS (AF4-MALS)   

Protein molar mass was measured using an AF4-MALS instrument (Wyatt Technology, Santa Barbara, California, USA). The protein buffer for full-length RsbV–RsbW, RsbV_1–104_–RsbW_5–145_, RsbW and RsbW–SigB was phosphate-buffered saline (10 m*M* Na_2_HPO_4_, 1.8 m*M* KH_2_PO_4_, 2.7 m*M* KCl, 0.137 *M* NaCl pH 7.4), whereas that for RsbV was buffer *A*. The Eclipse DualTec AF4-MALS system was equipped with a standard channel (246 mm in length), spacer (350 µm in thickness) and regenerated cellulose membrane (10 kDa cutoff). 50 µl of each protein was injected into the AF4-MALS system, which had been pre-equilibrated with protein buffer. The eluates were fractionated in the channel using an out-flow rate of 0.6 ml min^−1^ and a cross-flow rate of 2.5 ml min^−1^ and were detected by an 18-angle DAWN HELEOS II. Data were analyzed by fitting the experimental light-scattering data as a Zimm model and were plotted using an EASI graph with an RI peak using the *ASTRA* 6.1 software (Wyatt Technology).

### 2.4. SAXS   

SAXS data were collected on beamline 4C-SAXS II at Pohang Light Source II, Republic of Korea (PLS II-BL4C; Kim *et al.*, 2017 ▸). The light from the storage ring of Pohang Light Source II was focused on the beamline by a vertical focusing toroidal mirror coated with rhodium, and the X-rays had a wavelength of 0.734 Å, which was selected using a Si(111) double-crystal monochromator. The size of the X-ray beam exposed to the sample stage was 0.15 mm (vertical) × 0.24 mm (horizontal). Sample-to-detector distances were fixed at 100 and 400 cm, covering a scattering-vector magnitude range of 0.1 < *q* < 2.50 nm^−1^ (*q* = 4πsinθ/λ, where 2θ is the scattering angle and λ is the X-ray wavelength). Proteins at concentrations of 2.5 and 5.0 mg ml^−1^ were injected into quartz capillary cells (outside diameter 1.5 mm and wall thickness 0.01 mm). Scattering patterns were recorded on a two-dimensional SX-165 charge-coupled detector (Rayonix, Evanston, Illinois, USA) by exposure to X-rays for 10 s, and six consecutive scattering images were collected by running the protein solution at a flow rate of 0.3 µl s^−1^ through capillary cells using a Microlab 600 advanced syringe pump (Hamilton, Reno, Nevada, USA). Scattering angles were calibrated using polystyrene-b-polyethylene-b-polybutadiene-b-polystyrene block copolymer standards. The data were averaged radially and normalized to the intensity of the transmitted beam. The buffer scattering was subtracted from the protein scattering as background noise.

The radius of gyration (*R*_g,G_) was calculated by Guinier analysis of the scattering data (Strassburger *et al.*, 1982 ▸). The pair distance distribution function *p*(*r*) was calculated by indirect Fourier transform using *GNOM* (Semenyuk & Svergun, 1991 ▸). The SAXS parameters are summarized in Table 2 ▸. The *ab initio* molecular envelopes were reconstructed under a *P*22 symmetry restraint using *DAMMIF* (Franke & Svergun, 2009 ▸). The theoretical SAXS curve of the crystal structure was calculated using *CRYSOL* (Svergun *et al.*, 1995 ▸). To compare the overall shapes and dimensions, the crystal structure was superimposed onto reconstructed dummy-residue models using *SUPCOMB* (Kozin & Svergun, 2001 ▸).

## 3. Results   

### 3.1. Structure determination of the RsbV–RsbW complex   

*B. subtilis* RsbV and RsbW were co-expressed in *E. coli* and the complex was purified by nickel-affinity chromatography and SEC. Initially, the complex of full-length RsbV (residues 1–109) and RsbW (residues 1–160) was crystallized [Supplementary Fig. S1(*a*)], but the diffraction of this crystal was limited to 7 Å resolution. To improve the resolution limit, various truncated complexes of RsbV and RsbW were purified and crystallized. Of these, the complex of RsbV_1–104_ (residues 1–104) and RsbW_5–145_ (residues 5–145) formed plate-shaped monoclinic crystals [Supplementary Fig. S1(*b*)]. Diffraction data were collected at 3.4 Å resolution and the crystal structure was determined by MR using a homology model of the RsbV–RsbW complex as a template. Residues 59–64 and 85–113 in RsbW were not visible and were not modeled in the electron density. Overall, eight heterodimers of RsbV and RsbW were built in the asymmetric unit. The *R* and *R*_free_ values of the final model were 20.8% and 29.0%, respectively [Supplementary Fig. S2(*a*) and Table 1 ▸].

RsbV and RsbW are homologs of SpoIIAA and SpoIIAB, respectively [Supplementary Figs. S3(*a*) and S3(*b*)]. The ADP-bound form of the kinase SpoIIAB binds tightly to SpoIIAA (Duncan *et al.*, 1996 ▸). To understand the crystal structure of ADP-bound RsbW in complex with RsbV, we obtained rod-shaped hexagonal crystals of RsbV–RsbW in the presence of excess ADP [Supplementary Fig. S1(*c*)] and determined the crystal structure at 3.1 Å resolution. Two heterodimers of RsbV and RsbW were built in the asymmetric unit and the final model was refined to an *R* and *R*_free_ of 22.1% and 24.9%, respectively [Supplementary Fig. S2(*b*) and Table 1 ▸]. However, residues 86–109 of RsbW corresponding to the ADP-binding loop of *Geobacillus stearothermophilus* SpoIIAB [Supplementary Fig. S3(*b*)] were not visible in the electron-density map. The ADP molecule was also not observed in the map around the ADP-binding loop. Thus, the crystal structure of RsbV–RsbW was found to be an apo form that did not contain ADP, even though the RsbV–RsbW complex was crystallized in the presence of excess ADP.

### 3.2. Overall structures of RsbV and RsbW monomers   

**Figure 2:**
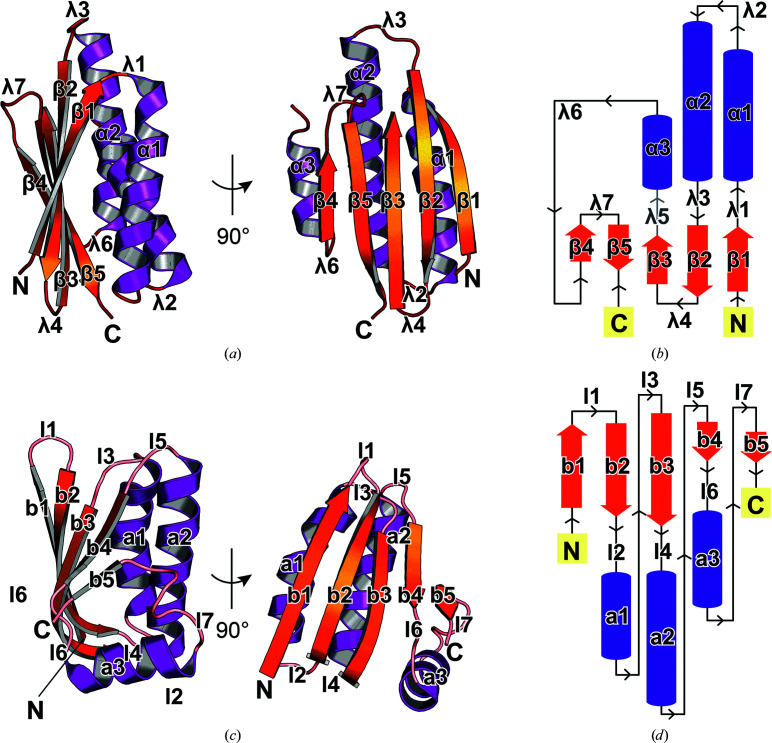
Overall structure of RsbW and RsbV. (*a*) Ribbon diagram and (*b*) topology model of the structure of RsbW. α-Helices, β-strands and loops are shown in purple, orange and red, respectively. The secondary structures are labeled α1–α3 for helices, β1–β5 for strands and λ1–λ7 for loops. λ5 between β3 and α3, which was not traced in the crystal structure, is shown in light gray. N and C represent the amino-terminus and carboxyl-terminus, respectively. (*c*) Ribbon diagram and (*d*) topology model of the structure of RsbV. The secondary structures are labeled a1–a3 for helices, b1–b5 for strands and l1–l7 for loops.

The RsbW monomer in the crystal structures of RsbV–RsbW exhibits a two-layered α/β fold. One layer forms an antiparallel β-sheet (β1–β5) and the other layer comprises three α-helices (α1–α3) [Figs. 2 ▸(*a*) and 2 ▸(*b*)]. The ADP-binding loop λ5 was not visible in the electron-density maps of both the monoclinic and hexagonal crystal structures [Fig. 2 ▸(*b*) and Supplementary Fig. S4]. The RsbW monomers in the asymmetric units of the monoclinic and hexagonal crystals are highly similar to each other. The root-mean-square deviation (r.m.s.d.) values between the RsbW monomers ranged between 0.4 and 0.8 Å. Compared with known structures, *G. stearothermophilus* SpoIIAB (PDB entry 1thn) showed the highest similarity. The r.m.s.d. values between the structures of SpoIIAB and RsbW ranged between 1.4 and 1.5 Å for 106 C^α^ atoms.

The RsbV monomer has the canonical α/β fold of STAS proteins, which consists of a superhelix of three β–α turns (b2–a1, b3–a2 and b4–a3) with additional β-strands (b1 and b5) at the N- and C-termini [Figs. 2(*c*) ▸ and 2(*d*) ▸]. The RsbV monomers in the crystal structure of RsbV–RsbW are also highly similar to each other. The r.m.s.d. values between the RsbV monomers from monoclinic and hexagonal structures ranged between 0.4 and 0.8 Å. Compared with known structures, *G. stearothermophilus* SpoIIAA (PDB entry 1thn) was most similar to RsbV. The r.m.s.d. values between the structures of SpoIIAA and RsbV ranged between 1.4 and 1.5 Å for 100 C^α^ atoms.

### 3.3. Structure of the RsbV–RsbW tetramer   

In the crystal structure of the SpoIIAA–SpoIIAB complex, SpoIIAB forms a homodimer and individual SpoIIAA monomers bind to the SpoIIAB monomers, forming a butterfly-shaped heterotetramer (Masuda *et al.*, 2004 ▸). The asymmetric units of the hexagonal and monoclinic crystals contain one and four heterotetramers of RsbV–RsbW, respectively. The structures of the RsbV–RsbW tetramer superimpose on those of the SpoIIAA–SpoIIAB tetramer with r.m.s.d. values of 1.9–2.1 Å for 394 C^α^ atoms, indicating that the tetrameric assembly of RsbV–RsbW is highly similar to that of SpoIIAA–SpoIIAB (Supplementary Fig. S5).

**Figure 3:**
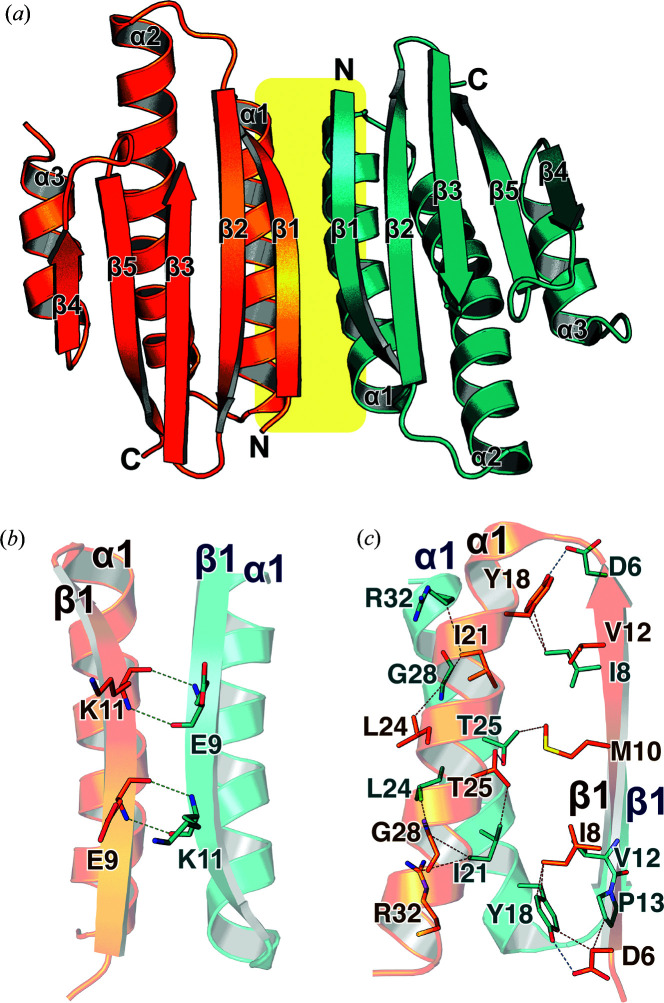
Structure of the RsbW homodimer. (*a*) Ribbon diagram of the RsbW homodimer. RsbW monomers are drawn as orange and cyan ribbon models. The dimer interface (surface-1) is shaded in yellow. (*b*, *c*) Surface-­1. Residues participating in dimerization are drawn as stick models. Red and blue dotted lines represent hydrogen bonds and hydrophobic interactions, respectively.

In the interface of the RsbW dimer (surface-1), the α1 helix and β1 strand directly mediate the dimerization, forming an extended α/β fold [Fig. 3 ▸(*a*)]. The β1 strands of two RsbW monomers form a cross β-sheet through backbone hydrogen bonds [Fig. 3 ▸(*b*)]. The α1 helix of the RsbW monomer interacts with the α1 helix and β1 strand of the other RsbW monomer in a symmetric manner [Fig. 3 ▸(*c*)]. The interactions in the monoclinic and hexagonal structures result in accessible surface-area burials of 748.5 and 786.5 Å^2^ and free-energy changes (Δ*G*) of −10.6 and −8.1 kcal mol^−1^, respectively, indicating that surface-1 is a biological binding interface.

**Figure 4:**
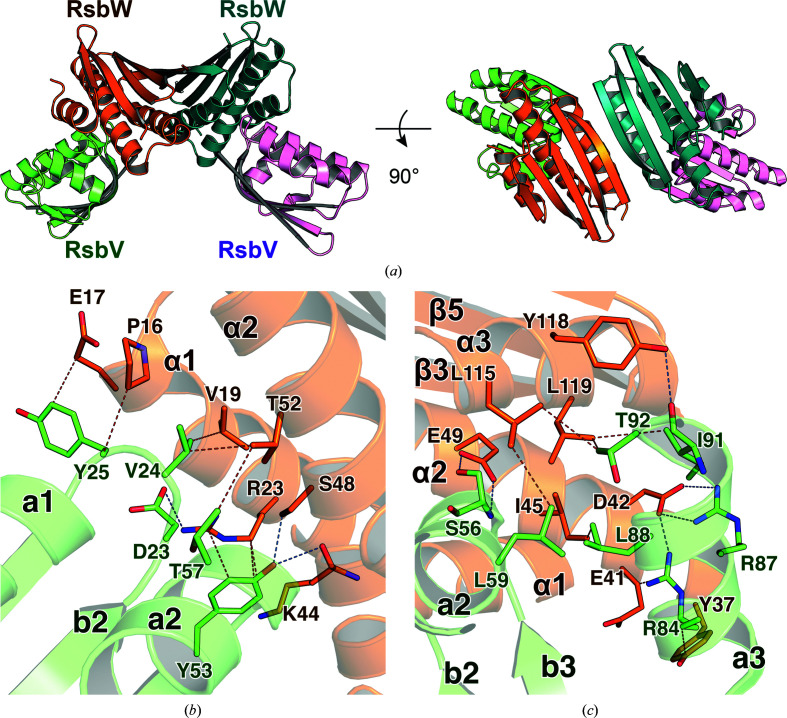
Structure of the RsbV–RsbW tetramer. (*a*) Overall structure of the RsbV–RsbW tetramer drawn as a ribbon diagram in two different orientations. Each monomer is colored differently. Two RsbV monomers individually bind to both sides of the RsbW homodimer. (*b*, *c*) Interactions between RsbV and RsbW. Residues of RsbV and RsbW that participate in the interaction are drawn as brown and pale green stick models, respectively. The red and blue dotted lines represent hydrogen bonds and hydrophobic interactions, respectively.

Two RsbV monomers bind to both sides of the RsbW dimer to form the RsbV–RsbW tetramer. Overall, three α-helices (α1–α3) in RsbW interact with the l2 loop, l4 loop and a3 helix of RsbV (Fig. 4 ▸). Arg23, Asp42, Lys44/Ser48, Glu49 and Tyr118 of RsbW form hydrogen bonds to Asp23, Arg84/Arg87, Tyr53, Ser56 and Ile91 of RsbV, respectively [Figs. 4 ▸(*b*) and 4 ▸(*c*)]. In the interactions, Glu49 of RsbW directly interacts with Ser56 of RsbV, which is phosphorylated by RsbW. The interactions between RsbV and RsbW in the monoclinic and hexagonal crystal structures result in accessible surface-area burials of 735.7 and 753.0 Å^2^ and Δ*G* values of −7.4 and −7.6 kcal mol^−1^ on average, respectively [Fig. 4 ▸(*a*)]. Overall, the RsbV–RsbW tetramers in the asymmetric units of the hexagonal and monoclinic crystals form the basic unit for complex assembly.

The conformation of the SpoIIAB dimer changes slightly when it binds SigF rather than SpoIIAA (Supplementary Fig. S5; Campbell *et al.*, 2002 ▸; Masuda *et al.*, 2004 ▸). When the RsbW dimer in the RsbV–RsbW tetramer was superimposed onto SpoIIAB, SpoIIAB in complex with SigF (1.3 Å for 213 C^α ^atoms) showed a lower r.m.s.d. value than that with SpoIIAA (1.8 Å for 210 C^α^ atoms). The angles between the α1 helix of one monomer and the α2 helix of the other monomer were calculated to be 54.9, 53.7 and 50.6° in the superimposed structures of RsbV–RsbW, SpoIIAB–SigF and SpoIIAB–SpoIIAA, respectively (Supplementary Fig. S5). These analyses indicate that the conformation of the RsbW dimer is closer to that of SpoIIAB–SigF than to that of SpoIIAA–SpoIIAB.

### 3.4. Octameric assembly of RsbV–RsbW   

**Figure 5:**
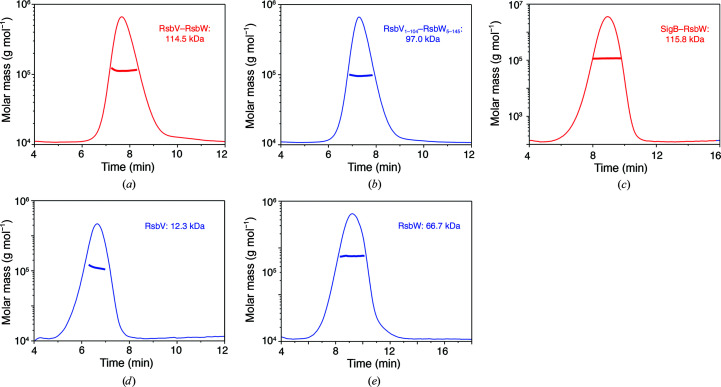
Assembly of RsbV–RsbW in solution. AF4-MALS profiles of (*a*) RsbV–RsbW, (*b*) RsbV_1–104_–RsbW_5–145_, (*c*) RsbW–SigB, (*d*) RsbV and (*e*) RsbW. The thick lines represent the molar masses of the proteins. All AF4-MALS profiles exhibit distinct single peaks, indicating that the protein solution does not contain any other oligomers or aggregates. The molecular masses of RsbV–RsbW and RsbV_1–104_–RsbW_5–145_ were calculated to be 114.5 and 97.0 kDa, respectively, which are close to the theoretical molecular masses of the hetero-octamers RsbV–RsbW (120.8 kDa) and RsbV_1–104_–RsbW_5–145_ (111.2 kDa). The molar masses of RsbV and RsbW were 12.3 and 66.7 kDa, indicating that RsbV and RsbW exist as monomers and tetramers in solution, respectively. The molar mass of RsbW–SigB was 115.8 kDa, which is close to that of an assembly of four RsbW and two SigB molecules.

SpoIIAA–SpoIIAB, which is homologous to RsbV–RsbW, forms a heterotetramer. In the crystal structures of RsbV–RsbW the conformation of the heterotetramer showed a significantly reduced free energy, indicating that the tetramer is a basic assembly unit of the RsbV–RsbW complex (Fig. 4 ▸). However, RsbV–RsbW was estimated to be an octamer by SEC (Supplementary Fig. S6). To confirm the oligomerization of RsbV–RsbW, we determined the molar mass of the complex using MALS. In AF4-MALS experiments, the molar mass of full-length RsbV–RsbW was calculated to be 114.5 kDa, which corresponds to an octamer that comprises four RsbV molecules and four RsbW molecules [Fig. 5 ▸(*a*)]. The molar mass of the truncated complex (RsbV_1–104_–RsbW_5–145_) used for structure determination was also close to the size of an octamer [97.0 kDa; Fig. 5 ▸(*b*)].

**Figure 6:**
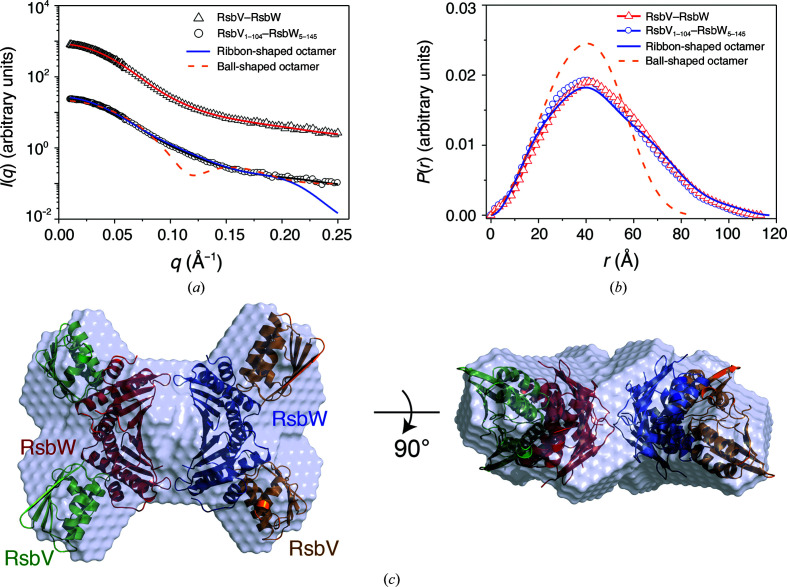
SAXS envelope structure of the RsbV–RsbW octamer. (*a*) X-ray scattering profiles of full-length RsbV–RsbW (triangles) and RsbV_1–104_–RsbW_5–145_ (circles). Red and black solid lines represent SAXS curves calculated from the SAXS envelope structures (χ^2^ = 0.077 for full-length RsbV–RsbW and 0.179 for RsbV_1–104_–RsbW_5–145_). The solid blue and dashed orange lines indicate theoretical SAXS curves calculated from ribbon-shaped and ball-shaped crystal structures, respectively (χ^2^ = 8.311 for the ball-shaped model and 0.812 for the ribbon-shaped model). For clarity, each curve is shifted along the log *I*(*q*) axis. (*b*) *p*(*r*) function profiles of full-length RsbV–RsbW (red triangles) and RsbV_1–104_–RsbW_5–145_ (blue circles). The solid blue and dashed orange lines indicate the theoretical *p*(*r*) function calculated from the ribbon-shaped and ball-shaped octamer models, respectively. The area under the curve was normalized to an equal area for easy comparison. (*c*) SAXS envelope structure of RsbV_1–104_–RsbW_5–145_. The SAXS envelope model was reconstructed under a *P*22 symmetry restraint and is superimposed with the ribbon-shaped crystal structure of the RsbV–RsbW octamer.

To confirm the octameric assembly of RsbV–RsbW, we performed SAXS experiments. The scattering data for RsbV–RsbW were collected by exposing protein solution flowing along a capillary tube to X-rays to minimize damage to the protein by X-rays, and multiple scattering images were averaged to reduce the background noise [Fig. 6 ▸(*a*)]. The scattering curves in a low-*q* region (Guinier region) fitted well to a straight line, indicating that RsbV–RsbW is in a monodisperse state without aggregation or inter-particle interference effects [Supplementary Fig. S7(*a*)]. The radius of gyration (*R*_g_) calculated from the slope of the linear fit within the Guinier region (*R*_g,G_) was slightly higher for RsbV–RsbW (34.90 Å) than for RsbV_1–104_–RsbW_5–145_ (32.21 Å), which is in agreement with the molecular size, indicating that the two RsbV–RsbW complexes are in the same oligomeric state (Table 2 ▸). The *p*(*r*) function, which is a histogram of distances between all possible pairs of atoms within a particle, was derived from the whole range of scattering data using indirect Fourier transformation. The maximum dimension (*D*_max_) of the *p*(*r*) function was 114.0 Å, and *R*_g,*p*(*r*)_ calculated from the *p*(*r*) function (35.91 Å) was correlated with the value obtained from the Guinier fit (*R*_g,G_) (Table 2 ▸). The *p*(*r*) function of RsbV_1–104_–RsbW_5–145_ was similar to that of RsbV–RsbW [Fig. 6 ▸(*b*)], whereas the values of *D*_max_ and *R*_g,*p*(*r*)_ were slightly lower than those of RsbV–RsbW, consistent with the molecular size (Table 2 ▸). The molar masses of RsbV–RsbW and RsbV_1–104_–RsbW_5–145_ estimated from the Porod volume were 133.6 and 108.7 kDa, respectively (Table 2 ▸), indicating that both RsbV–RsbW complexes form a hetero-octamer in solution, in agreement with the AF4-MALS result. Overall, the MALS and SAXS parameters showed that both RsbV–RsbW and RsbV_1–104_–RsbW_5–145_ exist in an octameric conformation without aggregation in solution.

Next, we searched for the unit of the RsbV–RsbW complex forming an octamer in the crystal structures. The asymmetric unit in the monoclinic crystal of the RsbV–RsbW complex contains two ball-shaped octamers, each of which is composed of two tetramer units [Supplementary Fig. S8(*a*)]. The b1 strand of RsbV mainly mediates the dimerization of the RsbV–RsbW tetramers [Supplementary Fig. S8(*b*)]. Asn4 and Val7 in the b1 strand of RsbV form hydrogen bonds to Asn/Asn17 and Lys33 in the other RsbV in a symmetric manner [Supplementary Fig. S8(*b*)]. The RsbV dimer is predicted to make crystallographic contacts with an accessible surface-area burial of 392.9 Å^2^ and an Δ*G* of 1.9 kcal mol^−1^. Another octamer unit is observed in both the monoclinic and hexagonal crystal structures. In the crystal structures, the RsbV–RsbW octamer forms a ribbon-shaped structure. RsbW forms a tetrameric core and binds four RsbV monomers, each of which lies outside the RsbW core [Supplementary Fig. S8(*c*)]. His57/Gly134 and His132 of RsbW form hydrogen bonds to His132 and His57/Gly134 of the other RsbW symmetrically, resulting in an accessible surface-area burial of 280.1 Å^2^ and a Δ*G* of −1.9 kcal mol^−1^ (surface-2) [Supplementary Fig. S8(*d*)]. Because the two binding interfaces of RsbW mediate the symmetric interaction, an accessible surface area of 560.1 Å^2^ is buried and the free energy is decreased by 3.8 kcal mol^−1^ on dimerization of the RsbW dimer. Overall, structural analyses showed that the ribbon-shaped octamer is more suitable than the ball-shaped octamer for biological assembly.

### 3.5. SAXS envelope structure of the RsbV–RsbW octamer   

The MALS and SAXS parameters clearly showed that RsbV–RsbW forms an octamer in solution (Figs. 5 ▸ and 6 ▸), and the crystal structure of RsbV–RsbW suggested that the ribbon-shaped assembly is a biological unit (Supplementary Fig. S8). In SAXS analysis, the *p*(*r*) function of RsbV–RsbW exhibits a near-symmetrical peak pattern with a tail, which is characteristic of a flattened globular conformation [Fig. 6 ▸(*b*)]. The *p*(*r*) function calculated from the crystal structure of the ribbon-shaped octamer fits well with those from the SAXS data of RsbV–RsbW, whereas the *p*(*r*) function from the ball-shaped octamer does not [Fig. 6 ▸(*b*)]. To visualize the envelope structure of RsbV–RsbW complexes, *ab initio* models were reconstructed from the SAXS data of RsbV–RsbW. Multiple models were built, and the most probable models were selected to improve the reliability of the final model. The reconstructed models of RsbV–RsbW and RsbV_1–104_–RsbW_5–145_ appear to be flat ribbon-shaped conformations, which overlap better with the crystal structure of a ribbon-shaped octamer rather than that of a ball-shaped octamer [Fig. 6 ▸(*c*) and Supplementary Fig. S7(*b*)]. The SAXS envelope structures revealed that RsbV–RsbW forms a ribbon-shaped octamer mediated by surface-2.

## 4. Discussion   

SigB is an alternative sigma factor that regulates transcription in *B. subtilis* in response to environmental and energy stresses. Its activity is inhibited by the direct binding of RsbW and is regulated by the binding-partner switching of RsbW to SigB or RsbV. In the present study, the crystal structures and SAXS envelope structure clearly showed that RsbV and RsbW assemble into an octamer in solution. Initially, the crystal structure of the RsbV–RsbW complex showed the formation of a butterfly-shaped tetramer with a significant reduction in free energy (Figs. 3 ▸ and 4 ▸). The tetrameric form coincides with previous reports showing that RsbW predominantly exists as a homodimer in solution (Dufour & Haldenwang, 1994 ▸; Delumeau *et al.*, 2002 ▸), while SpoIIAA–SpoIIAB, which is homologous to RsbV–RsbW, forms a stable heterotetramer. However, RsbV and RsbW were calculated to be a monomer and a tetramer, respectively, in monodisperse forms in MALS experiments (Fig. 5 ▸). The tetrameric assembly of RsbW was not changed on binding to RsbV or SigB, indicating that RsbW forms the stable core of a homotetramer (Fig. 5 ▸). Consistently, structural analyses of RsbV–RsbW, supported by SAXS and MALS experiments, revealed that the unique octameric assembly of RsbV–RsbW was mediated by RsbW. RsbV–RsbW was calculated to be an octamer in solution, and a ribbon-shaped octamer in the crystal structures of RsbV–RsbW overlaps well with the SAXS envelope structure (Figs. 5 ▸ and 6 ▸ and Supplementary Fig. S8). In the crystal structure of the RsbV–RsbW octamer, the two surface-2s strengthen the dimerization of the RsbV–RsbW tetramers by widening the binding area, although a single surface-2 is not sufficient to mediate the assembly [Supplementary Figs. S8(*c*) and S8(*d*)].

**Figure 7:**
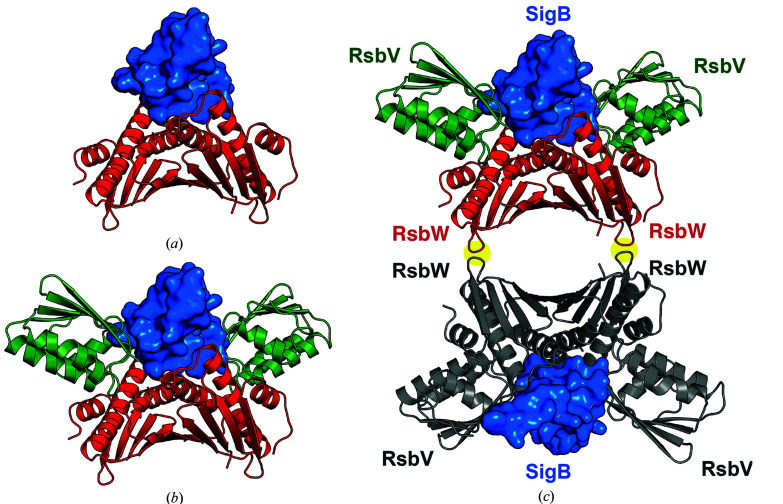
Model of SigB regulation by RsbV and RsbW. (*a*) Model structure of the RsbW dimer in complex with SigB. The model was prepared by superimposing the RsbW dimer and the SigB homology model on the structure of the SpoIIAA–SigF complex. The RsbW dimer and SigB are drawn as a red ribbon diagram and a blue surface model, respectively. (*b*) Model structure of RsbW–SigB superimposed on the crystal structure of the RsbV–RsbW tetramer. In addition to (*a*), RsbV is drawn as a green ribbon model. In the model, SigB clashes with RsbV when they bind to RsbW, indicating that binding of RsbV and SigB to RsbW is mutually exclusive. (*c*) Model structure of the RsbW–SigB hexamer superimposed on the crystal structure of the RsbV–RsbW octamer. Surface-2 mediating dimerization of the RsbW dimer is shaded with yellow circles.

The anti-anti-sigma factor RsbV, dephosphorylated under stress signals, releases SigB from the RsbW–SigB complex by hijacking the anti-sigma factor RsbW in a mutually exclusive manner with SigB (Dufour & Haldenwang, 1994 ▸; Voelker *et al.*, 1996 ▸; Delumeau *et al.*, 2002). The mechanism by which anti-sigma and anti-anti-sigma factors regulate the activity of sigma factors, known as partner switching of the anti-sigma factor, is well established in the regulation of *B. subtilis* SigF. SigF is activated in the early stage of *B. subtilis* sporulation (Margolis *et al.*, 1991 ▸) and is regulated by the anti-sigma factor SpoIIAB and the anti-anti-sigma factor SpoIIAA (Yudkin & Clarkson, 2005 ▸). In the crystal structures of SpoIIAA–SpoIIAB (Masuda *et al.*, 2004 ▸) and SpoIIAA–SigF (Campbell *et al.*, 2002 ▸), a single SigF molecule and two SpoIIAA molecules are placed in the middle and on both sides of the SpoIIAB dimer, respectively. When the SpoIIAB dimers of the two structures were superimposed, a structural clash and electrostatic repulsion occurred because of the negatively charged residues Glu21 in SpoIIAA and Asp148 in SigF (Masuda *et al.*, 2004 ▸). As ADP-bound SpoIIAB binds non­phosphorylated SpoIIAA with a higher affinity than SigF (Duncan *et al.*, 1996 ▸), SigF is released from SpoIIAB in the presence of nonphosphorylated SpoIIAA (Duncan *et al.*, 1996 ▸; Alper *et al.*, 1994 ▸). The residues for the binding of the sigma factor and anti-anti-sigma factor, as well as the overall structures, are conserved between RsbW and SpoIIAB (Supplementary Figs. S3 and S5). Moreover, RsbW interacts with SigB in a 2:1 ratio like SpoIIAB and SigF [Fig. 5 ▸(*c*)] (Delumeau *et al.*, 2002 ▸). Thus, the mechanisms by which anti-sigma and anti-anti-sigma factors regulate the activity of sigma factors may be similar in the two sigma/anti-sigma/anti-anti-sigma systems SigF–SpoIIAB–SpoIIAA and SigB–RsbW–RsbV. Notably, the residues at the clash point between the sigma factor and anti-anti-sigma factor under anti-sigma factor binding are conserved as negatively charged residues (Glu21 in RsbV and Glu148 in SigB; Supplementary Fig. S3). In the model structure of the RsbW–SigB complex, SigB is placed in the middle of the RsbW dimer [Fig. 7 ▸(*a*)]. Although the RsbW surfaces for the binding of RsbV and SigB do not overlap, the structures of RsbV and SigB clash with each other on RsbW binding [Fig. 7 ▸(*b*)], indicating that the binding of RsbV and SigB to RsbW is mutually exclusive. In the crystal structure of RsbV–RsbW, the RsbW dimer is additionally dimerized to form a homotetramer. Thus, four RsbV monomers or two SigB monomers are assembled onto a tetrameric core of RsbW in a mutually exclusive manner [Fig. 7 ▸(*c*)]. An increased understanding of the assembly is necessary to determine whether the RsbW core is required for the allosteric regulation of SigB activity.

## Supplementary Material

Supplementary Figures. DOI: 10.1107/S2052252520007617/lz5037sup1.pdf

PDB reference: *B. subtilis* RsbV–RsbW complex, monoclinic crystal form, 6m36

PDB reference: hexagonal crystal form, 6m37

## Figures and Tables

**Table 1:** Data-collection and refinement statistics for the structure determination of RsbV–RsbW Values in parentheses are for the highest resolution shell.

	Monoclinic RsbV–RsbW	Hexagonal RsbV–RsbW
Data collection		
X-ray source	PLS II-BL11C	PLS II-BL11C
Space group	*P*2_1_	*P*6_2_22
*a*, *b*, *c* (Å)	117.23, 70.94, 137.68	158.20, 158.20, 96.60
α, β, γ (°)	90.00, 105.35, 90.00	90.00, 90.00, 120.00
Resolution (Å)	50.0–3.40 (3.61–3.40)	50.0–3.10 (3.31–3.10)
Wavelength (Å)	0.97933	0.97933
Total/unique reflections	91522/29346 (13674/4589)	127495/13426 (22682/2377)
Completeness (%)	96.7 (94.7)	99.9 (100.0)
〈*I*/σ(*I*)〉	6.8 (2.4)	15.3 (3.6)
*R*_merge_ (%)	14.7 (49.2)	9.0 (65.4)
Refinement		
Resolution (Å)	30.0–3.40	50.0–3.10
No. of reflections		
Working	27753	12750
Free	1465	653
*R*_work_/*R*_free_ (%)	20.8/29.0	22.1/24.9
No. of atoms	12580	3330
R.m.s.d.		
Bond lengths (Å)	0.011	0.011
Bond angles (°)	1.447	1.428
Ramachandran plot (%)		
Favored	96.0	96.6
Allowed	3.8	2.9
Disallowed	0.2	0.5

**Table 2:** Structural parameters obtained from the SAXS data of proteins in solution

	*R*_g,G_ † (Å)	*R*_g,*p*(*r*)_ ‡ (Å)	*D*_max_ § (Å)	Vp¶ (Å^3^)	MM_calculated_ †† (kDa)	MM_SAXS,Vp_ ‡‡ (kDa)	Conformer
RsbV–RsbW	34.90 ± 0.30	35.91 ± 0.25	114.0	161000	30.2	133.6	Hetero-octamer
RsbV_1–104_–RsbW_5–145_	32.21 ± 0.29	34.66 ± 0.22	108.0	131000	27.8	108.7	Hetero-octamer
Ball-shaped octamer	30.04 ± 0.01	29.55 ± 0.07	85.0	115000	22.4		Hetero-octamer
Ribbon-shaped octamer	35.87 ± 0.01	36.13 ± 0.07	117.0	137000	22.4		Hetero-octamer

† Radius of gyration obtained from the scattering data by Guinier analysis.

‡ Radius of gyration obtained from the *p*(*r*) function by *GNOM*.

§ Maximum dimension obtained from the *p*(*r*) function by *GNOM*.

¶ Porod volume obtained from the *p*(*r*) function by *GNOM*.

†† Molecular mass obtained from the amino-acid sequence of the heterodimeric protein.

‡‡ Molecular mass calculated by multiplying Vp by the average protein density ρ_m_ = 0.83 × 10^−3^ kDa Å^−3^.
