# Supplementary material for: Structural insights into the regulation of SigB activity by RsbV and RsbW
Source: IUCrJ. 2020 Jun 24;7(Pt 4):737–47. doi: 10.1107/S2052252520007617 (PMC7340262; doi:10.1107/S2052252520007617)
Supplement: Supplementary file 1 [file m-07-00737-sup1.pdf]

IUCrJ

Volume 7 (2020)

Supporting information for article:

Structural insights into the regulation of SigB activity by RsbV and RsbW

Deepak Pathak, Kyung Sik Jin, Sudarshan Tandukar, Jun Ha Kim, Eunju Kwon and Dong Young Kim

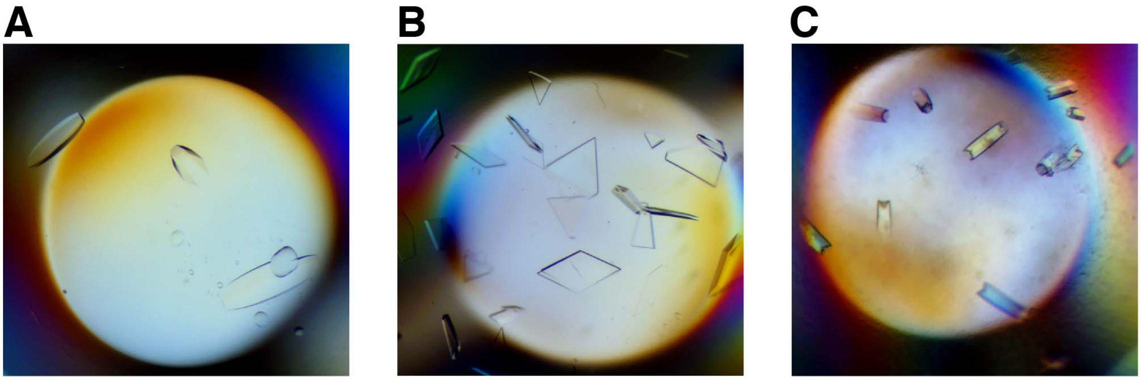

Figure S1 Crystals of RsbV/RsbW complex. (*a*) Oval-shaped crystals of full-length RsbV/RsbW. (*b*) Plate and (*c*) rod crystals of $RsbV_{1-104}/RsbW_{5-145}$.

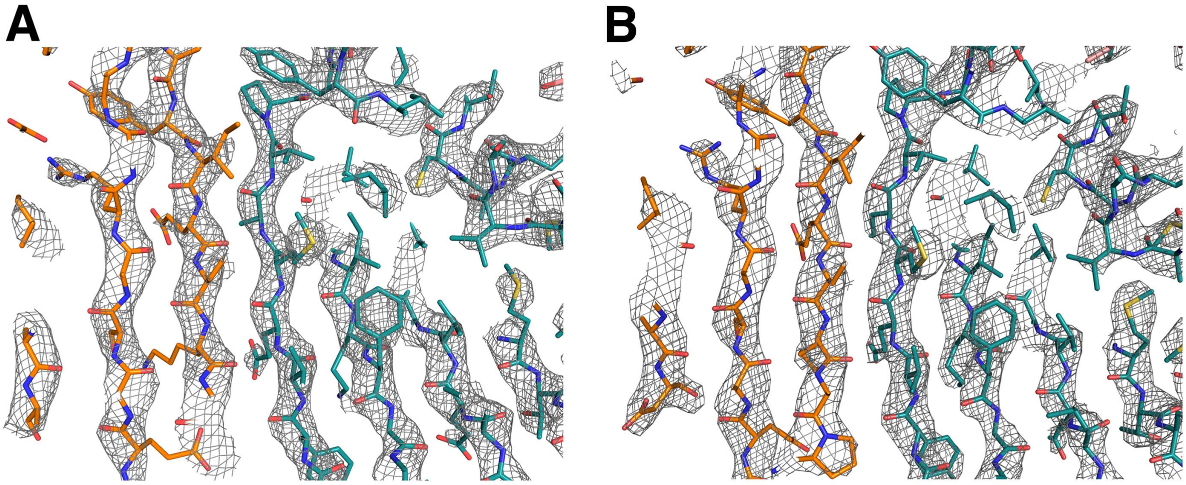

Figure S2 2Fo-Fc electron density maps of the RsbV/RsbW complex. (*a, b*) Electron density maps near the dimer interface of RsbV and RsbW. RsbV and RsbW are drawn as cyan and orange stick models, respectively. The electron density maps of monoclinic crystal and hexagonal crystal structures are displayed at 1.5 contour levels in (*a*) and (*b*), respectively.

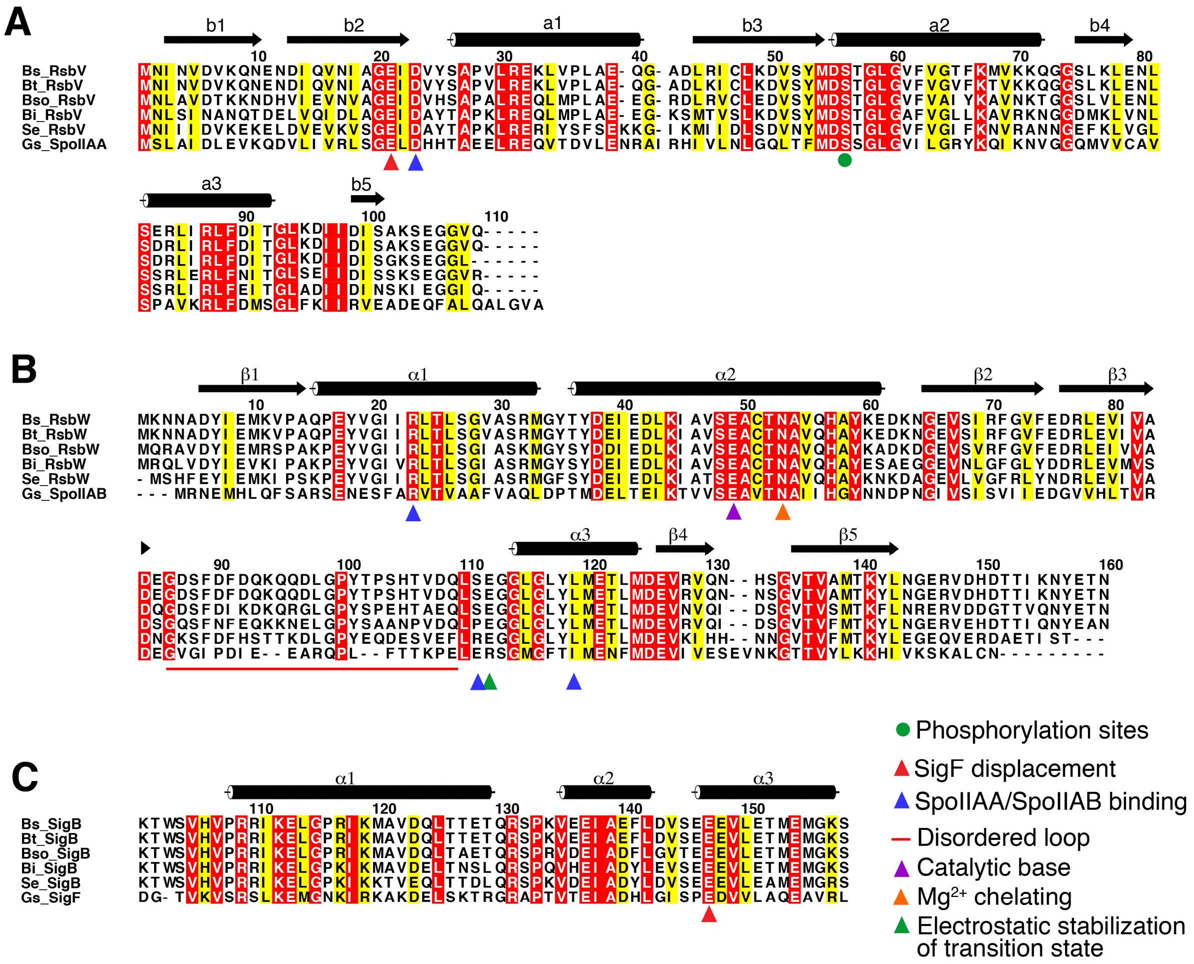

Figure S3 Sequence alignments. (*a*) Sequence alignment of RsbV and homologs. (*b*) Sequence alignment of RsbW and homologs. (*c*) Sequence alignment of RsbW binding motif in SigB and its homologs. Arrows and tubes indicate β-strands and tubes, respectively. The abbreviations of the species are: Bs, *Bacillus subtilis*; Bt, *Bacillus tequilensis*; Bso, *Bacillus sonorensis*; Bi, *Bacillus indicus*; Se, *Salmonella enterica*; Gs, *Geobacillus stearothermophilus*.

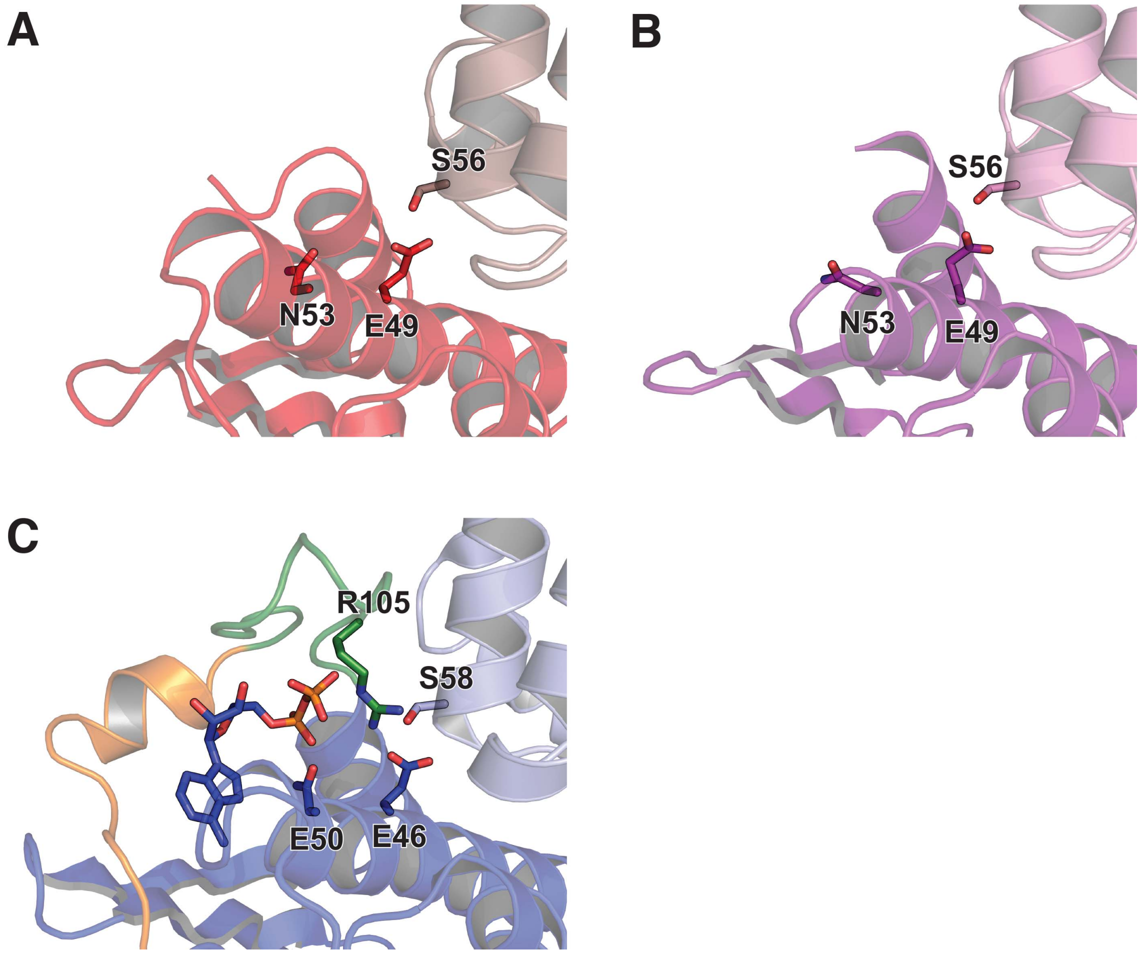

Figure S4 Structures near adenine-nucleotide binding loop. (*a*) Monoclinic crystal structure of RsbV/RsbW, (*b*) hexagonal crystal structure of RsbV/RsbW, and (*c*) crystal structure of SpoIIAA/SpoIIAB are drawn as ribbon models in the same orientations.

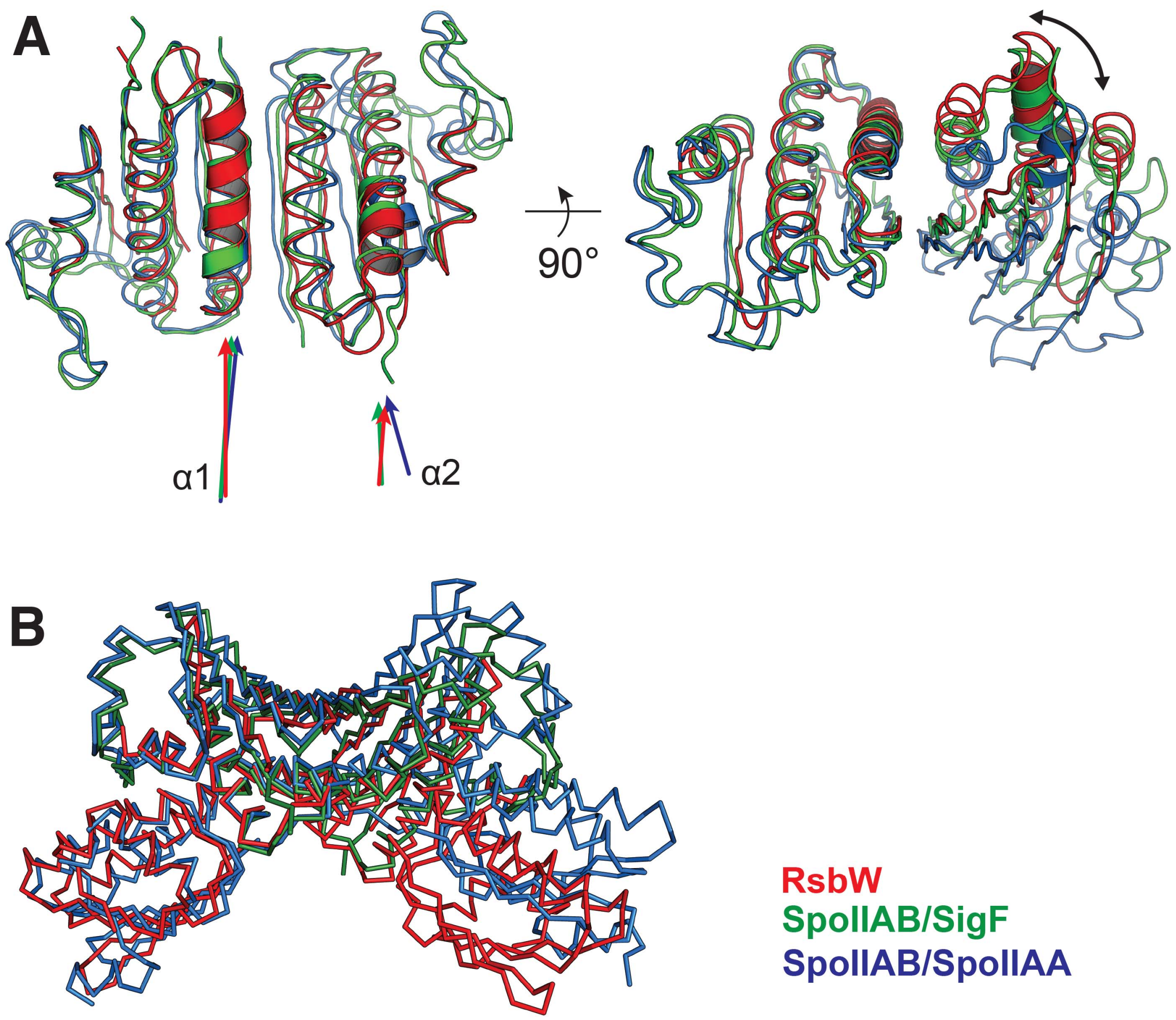

Figure S5 Structure of the RsbV/RsbW tetramer. (*a*) Structural comparison of RsbW and SpoIIAB homodimers. RsbW dimer and SpoIIAB dimers from the crystal structures of RsbV/RsbW, SpoIIAB/SigF, and SpoIIAB/SpoIIAA were superimposed onto each other, based on a monomer structure. Red, blue, and green models represent the RsbW dimer, SpoIIAB dimer from the crystal structure of SpoIIAA/SpoIIAB, and SpoIIAB dimer from the crystal structure of SpoIIAB/SigF, respectively. (*b*) Ca traces of the RsbV/RsbW tetramer (red), SpoIIAA/SpoIIAB tetramer (blue), and SpoIIAB dimer from crystal structure of SpoIIAB/SigF (green).

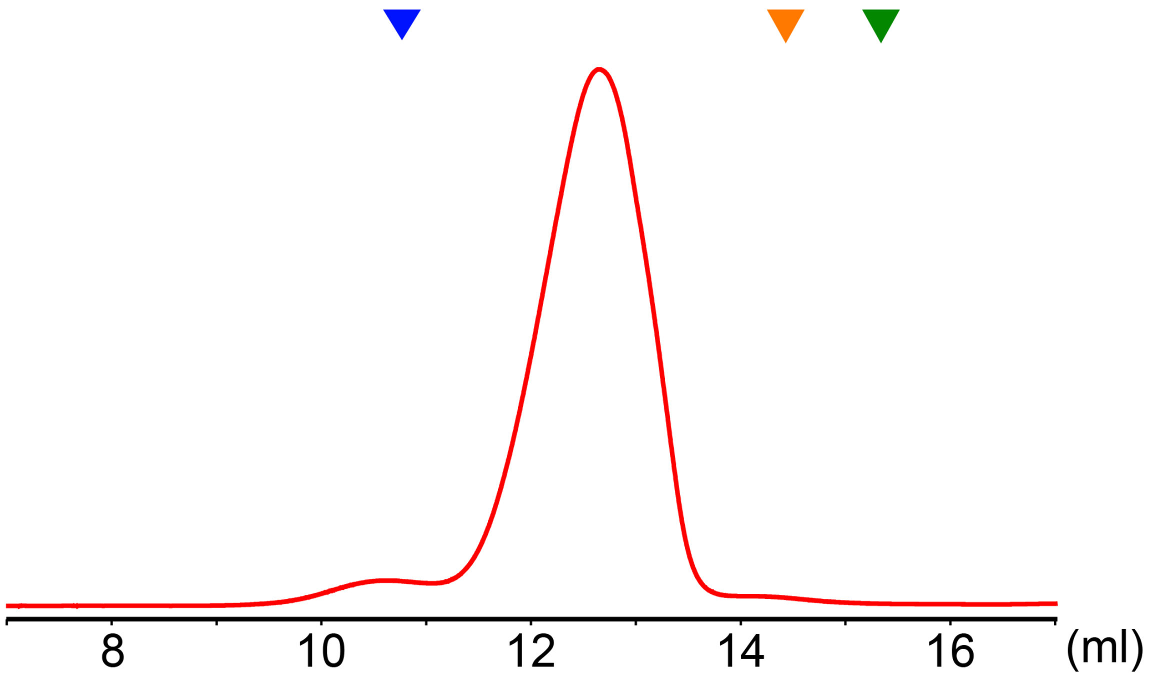

Figure S6 SEC of the RsbV/RsbW complex. Blue, orange, and green triangles indicate the positions of UV peaks of ferritin (molar size 440 kDa; elution volume 10.8 mL), conalbumin (76 kDa; 14.4 mL), and ovalbumin (43 kDa; 15.3 mL) eluted from a superdex200 analytical column. RsbV/RsbW was eluted between ferritin and conalbumin (elution volume 12.7 mL).

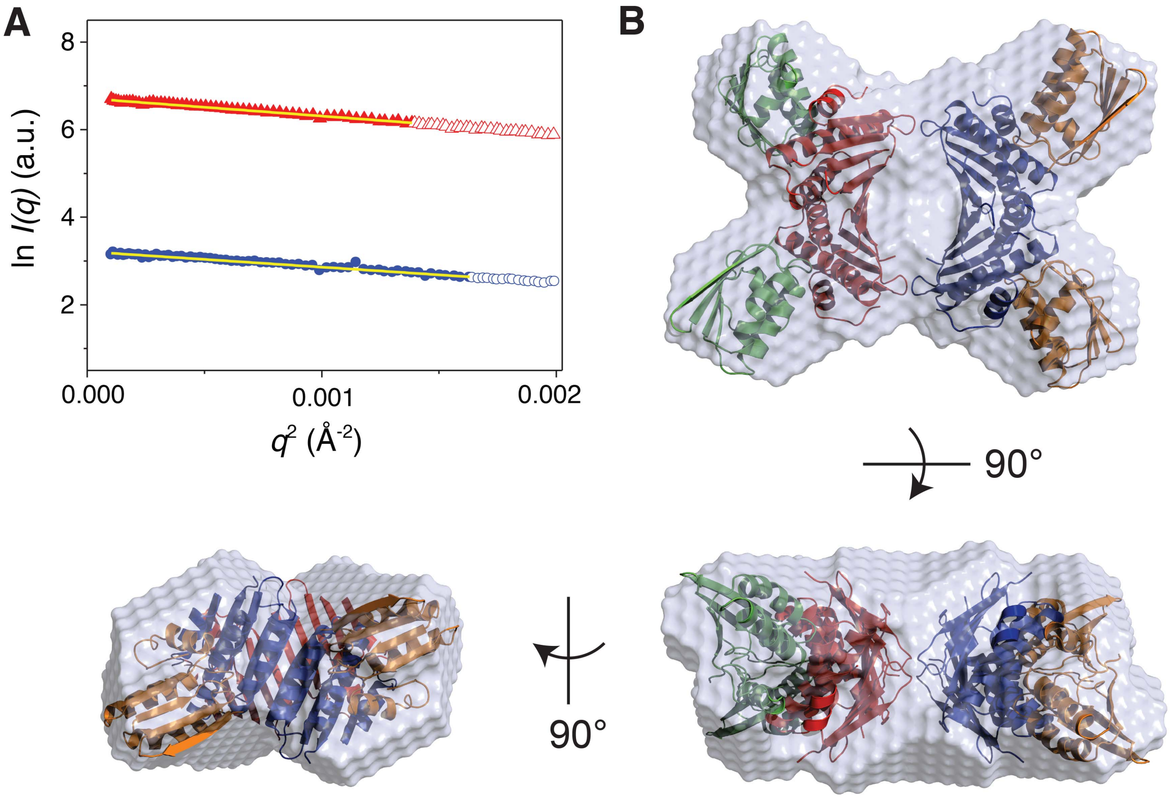

Figure S7 SAXS. (*a*) Guinier plots of the scattering profiles of full-length RsbV/RsbW and RsbV$_{1-104}$/RsbW$_{5-145}$. The Guinier fits (yellow lines) were obtained from the scattering data in the q^2 region for $qR_g < 1.3$. Open symbols indicate data outside of the Guinier region. Each plot is shifted along the ln $I(q)$ axis for clarity. (*b*) SAXS envelope structure of full-length RsbV/RsbW. Crystal structure of a ribbon-shaped RsbV/RsbW octamer was overlaid with the reconstructed DAMMIF dummy model to compare overall shape and dimensions (NSD = 1.984).

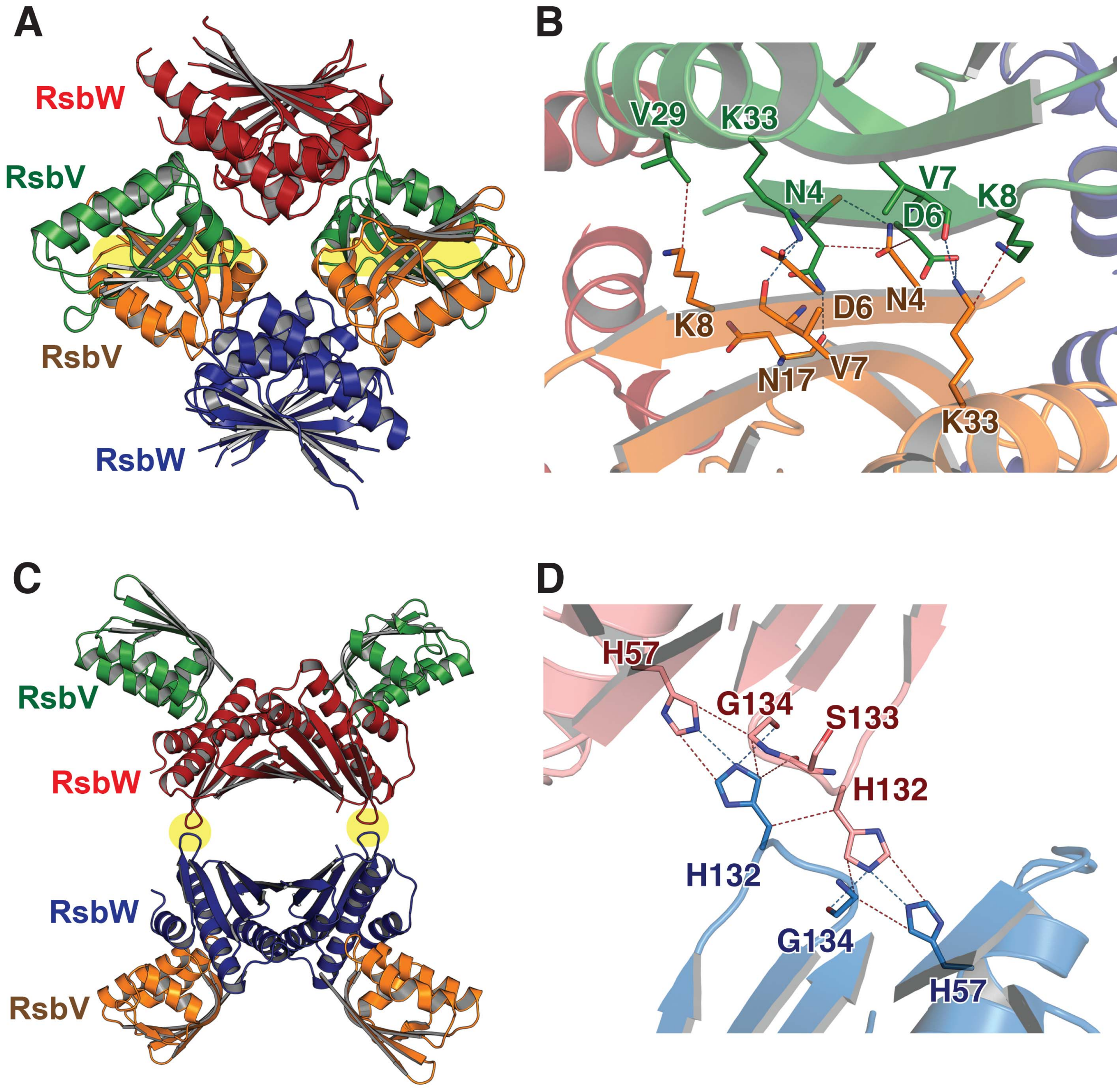

Figure S8 Crystal structures of the RsbV/RsbW octamer. (*a*) Ball-shaped octamer structure. Interface that mediates dimerization of RsbV/RsbW tetramers is indicated in yellow. (*b*) Interactions in the dimeric interface. Two RsbV monomers are drawn as green and orange ribbon models. Residues that mediate interactions are shown as stick models. Hydrogen bonds and hydrophobic interactions are indicated as blue and red dotted lines, respectively. (*c*) Ribbon-shaped octamer structure. The octamer assembly is mediated by surface-2 of RsbW shaded with yellow circle. (*d*) Interactions in surface-2. Two RsbW monomers are drawn as light red and light blue ribbon models. Residues that mediate the interactions are shown as stick models.
